# The Proper Way to Sit

**Published:** 1892-08

**Authors:** 


### THE PROPER WAY TO SIT.

A proper sitting position requires that the spine shall be kept straight and that the support needed for the upper part of the body shall be felt in the right place. Therefore, sit as far back as possible in the chair, so that the lower end of the spine shall be braced against the back of the seat. If this back is straight the shoulders will also rest against it; if not, they will have no point of support, and it will be found that they do not need it. This position makes no strain upon the ligaments of the spine. It allows a proper position of the shoulders, consequently of the chest, consequently of the lungs, stomach and every other organ of the body.

Their work is carried on naturally and comfortably, as is also the circulation of the blood, which in a wrong sitting position is seriously interfered with. With the feet resting squarely upon the floor, the hands resting easily upon the lap, perfect equilibrium, and consequently perfect rest of the body is secured. There is no strain upon any part of the body; no muscle or organ is required to do more than its legitimate amount of work. The arms should never be folded, for that position not only causes a strain upon the spine, and all the other evils already referred to, but, in addition, places the weight of the arms upon the stomach and the diaphragm, thereby increasing the labor of digestion and respiration. Placing the hands behind the back, if possible, is a good attitude to take occasionally, giving as it does, the fullest expansion of the whole upper part of the body.

